# Shape-memory surfaces for cell mechanobiology

**DOI:** 10.1088/1468-6996/16/1/014804

**Published:** 2015-02-18

**Authors:** Mitsuhiro Ebara

**Affiliations:** Biomaterials Unit, International Center for Materials Nanoarchitectonics (WPI-MANA), Institute for Materials Science (NIMS), 1-1 Namiki, Tsukuba, Ibaraki 305-0044, Japan

**Keywords:** shape-memory polymers, shape-memory surfaces, cell mechanobiology

## Abstract

Shape-memory polymers (SMPs) are a new class of smart materials, which have the capability to change from a temporary shape ‘A’ to a memorized permanent shape ‘B’ upon application of an external stimulus. In recent years, SMPs have attracted much attention from basic and fundamental research to industrial and practical applications due to the cheap and efficient alternative to well-known metallic shape-memory alloys. Since the shape-memory effect in SMPs is not related to a specific material property of single polymers, the control of nanoarchitecture of polymer networks is particularly important for the smart functions of SMPs. Such nanoarchitectonic approaches have enabled us to further create shape-memory surfaces (SMSs) with tunable surface topography at nano scale. The present review aims to bring together the exciting design of SMSs and the ever-expanding range of their uses as tools to control cell functions. The goal for these endeavors is to mimic the surrounding mechanical cues of extracellular environments which have been considered as critical parameters in cell fate determination. The untapped potential of SMSs makes them one of the most exciting interfaces of materials science and cell mechanobiology.

## 1. Introduction

Over the past few decades, increased attention has been given to smart polymers, also termed stimulus-responsive polymers, owing to their ability to act as an ‘on–off’ reversible switch in response to small changes in their environment [1]. The affected factors include shape [2], surface characteristics [3], solubility [4], molecular affinity [5], and sol–gel transition [6], etc, whereas the external triggers can be temperature [7], pH [8], ionic strength [9], certain chemicals [10], electric field [11], magnetic field [12], or light [13]. Because such smart materials mimic the smartness of biological systems in the body (e.g., variations of pH along the gastrointestinal tract, homeostatic regulation system, or blood coagulation system), smart polymer-based technologies have been investigated and developed for a variety of medical, biological, and analytical technologies. For example, Garbern *et al* have reported pH-responsive injectable hydrogels for delivering drugs to regions of local acidosis such as ischemic or infected sites [14]. This pH-responsive system has been also shown to enhance the cytosolic delivery of nucleic acids [15]. These unique characteristics of smart polymers, on the other hand, offer an opportunity for developing remote-controllable systems on demand. For example, Shimoboji *et al* developed light-responsive polymer–enzyme switches and demonstrated ‘on–off’ control of the catalytic activity by alternating irradiation with UV and visible light [16]. The author’s group has recently developed a smart hyperthermia nanofiber with switchable heat generation and drug release by remote-controlling of alternating magnetic field for cancer therapy [17].

Among these smart polymers, shape-memory polymers (SMPs) have emerged as a new class of smart polymers that have the capability to change from a temporary shape to a memorized permanent shape upon application of an external stimulus. SMPs also represent a cheap and efficient alternative to well-known metallic shape-memory alloys (SMAs) because they are relatively easy to manufacture and program. The use of SMPs as self-repairing or rewritable materials has found growing interest in environmentally friendly technologies. The history of SMPs goes back to the 1940s. The first mention of SMPs is in a United States patent applied by Vernon and Vernon in 1941 [18, 19]. They developed dental restoration materials with elastic memory property, which are thermoplastic synthetic resins made of methacrylic acid ester. Since then, many efforts to design and develop SMPs have started. Especially, the 1980s was considered as a time period when SMP research was at its first peak. The term SMPs became well known with the discovery of poly(norbornene), which was developed by CDF-Chimie and commercialized by Nippon Zeon using the brand name Norsorex in the 1980s. Other types of SMPs, such as TP-301 based on poly(trans-isoprene) and Asmer based on poly(styrene-butadiene), were subsequently developed by two other Japanese companies (Kurare Corporation and Asahi Kasei Corporation, respectively) [20, 21]. The segmental polyurethane (PU)-based SMPs were also developed by Mitsubishi Heavy Industries in the early 1990s [22–25]. Thus, the earlier works on SMPs were mainly in industrial fields. Significant research efforts started again in the early 2000s after Lendlein and Langer demonstrated the concept of biodegradable thermally responsive SMP sutures [26]. Since then, many researchers have tried to introduce biocompatibility and biodegradability into SMPs for various biomedical applications which has led to substantially improved health care, such as implantable devices or minimally invasive surgical procedures without damaging the surrounding tissues/organs. In addition, SMPs have also presented additional potential in the area of micro-electro-mechanical systems (MEMS), actuators, self-healing, unique adhesion devices and cell culture platforms [27–29]. Because the surface morphology is of fundamental important to many surface-related material properties, recent studies started to use SMPs as substrates to form topography with tunable property, namely shape-memory surfaces (SMSs).

**Figure 1.**
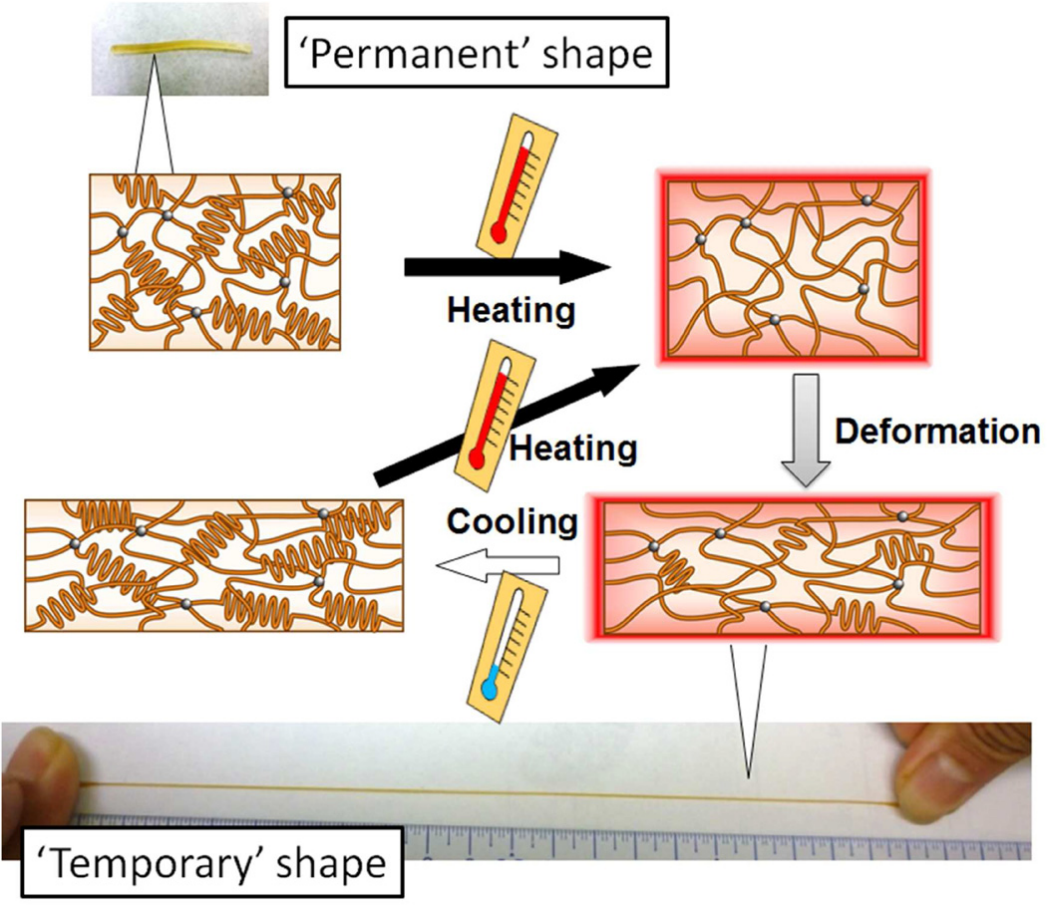
Schematic representation of the mechanism of shape-memory effects for thermally induced shape-memory polymers (SMPs) based on a crystal-amorphous transition in a semicrystalline-based polymer network.

**Figure 2.**
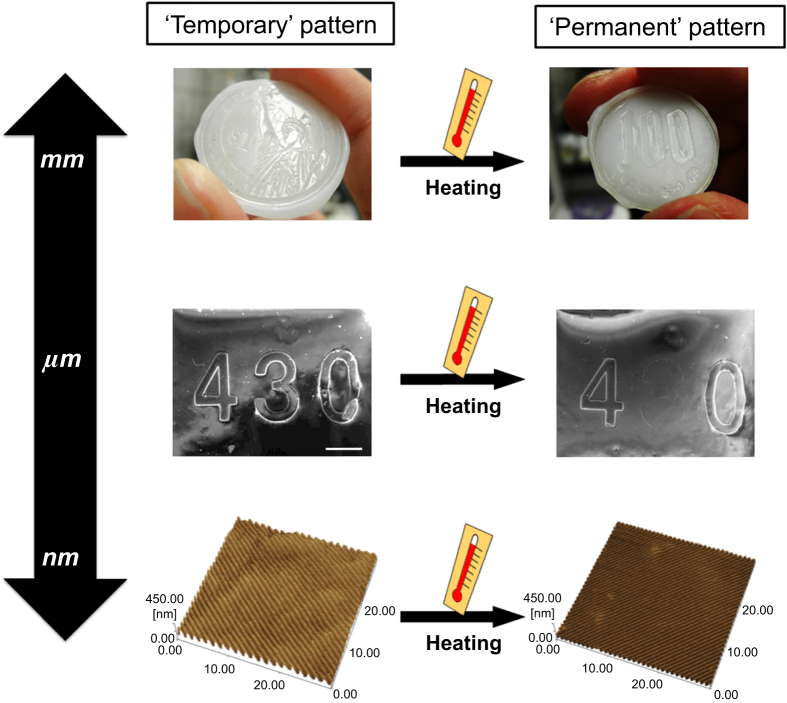
Surface shape-memory transition from a memorized ‘temporary’ pattern (left) to the original ‘permanent’ pattern (right).

Figure 1 shows the schematic images of thermally induced crystal/amorphous transition in SMPs. While the origin of the shape-memory effect in SMAs is the solid-phase transition between the austenite phase at higher temperature and the martensite phase at lower temperature, that in SMPs relies on the phase transformation of switching domains or segments, such as glass–rubber and/or crystal–amorphous transitions [30]. The most noteworthy and extensively researched group of SMPs is thermally induced SMPs. They are thermoplastic elastomers or thermosets that are programmed by mechanically deforming the shape of a polymer at a temperature that exceeds its glass transition temperature (*T*_g_) or melting temperature (*T*_m_). This deformed shape (or ‘temporary’ shape) can be fixed when the material is cooled below the *T*_g_ or *T*_m_. If the polymer chains are chemically or physically cross-linked, the material returns to its original shape (or ‘permanent’ shape) by heating it above the *T*_g_ or *T*_m_. During this process, an increase in entropy serves as the driving force for the material to recover its initial shape. Therefore, shape-memory effects of thermally induced SMPs can be precisely controlled by tailoring the nano-architectures of polymer networks such as crystallinity or crosslinking density. Such nano-architectonic approaches have enabled us to develop SMPs with a thermal switch at a biologically relevant temperature [31–34]. Moreover, recent studies have shown the successful memorization of ordered patterns with feature sizes on the submicron scale to create SMSs (figure 2) [35–37]. The SMSs can offer an elegant approach to fabricating cell culture substrates with dynamically tunable topographies for investigating cell mechanobiology. The area of cell mechanobiology has recently been the subject of active research because the cell–extracellular matrix (ECM) interactions are considered to be important extrinsic factors that regulate cell fate. The manipulation of topographical cues, therefore, has a strong potential as a strategy for stem cell engineering and regenerative medicine by mimicking changing physiological conditions during would healing, organogenesis, and cancer metastasis. In this review, different types of SMPs (especially thermally induced SMPs) are discussed on the basis of nanoarchitectures of polymer networks. In addition, recent progresses of SMPs in biotechnology and biomedicine are reviewed. Especially, this review focuses on the developments of SMSs with dynamically switchable topography to control cell–substrate interactions and to direct cell fate.

## 2. Classification of SMPs on the basis of nanoarchitechtonics

**Figure 3.**
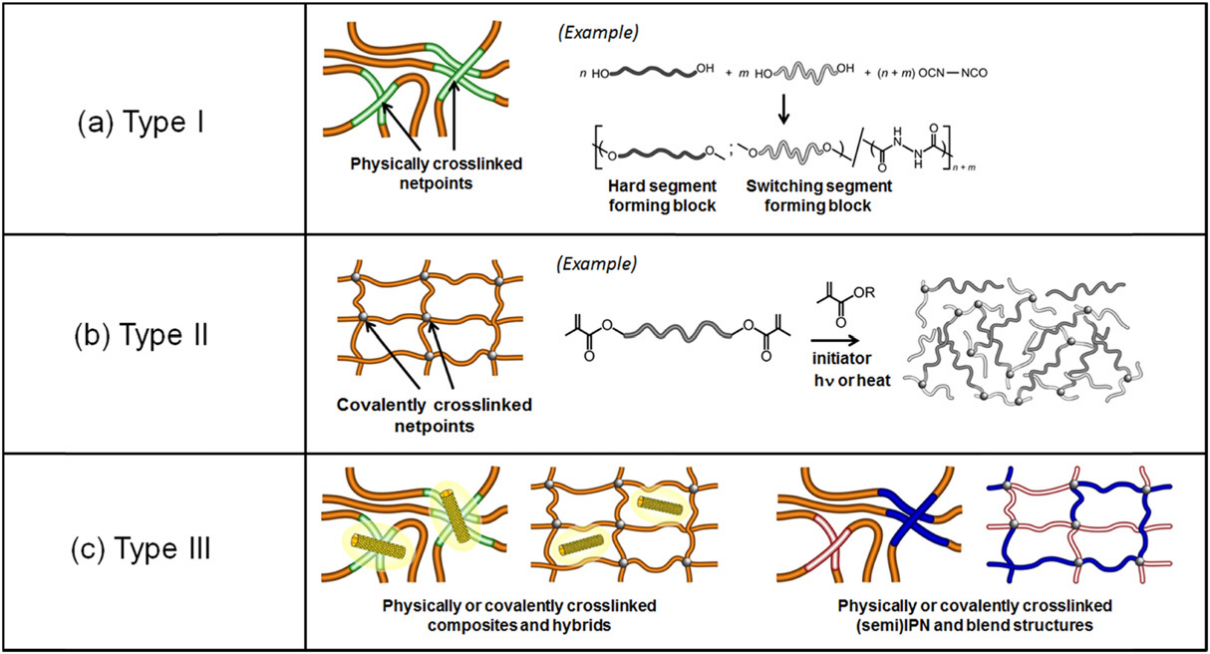
Classification on the basis of types of polymer network architecture: physically cross-linked (type I) and covalently cross-linked SMPs (type II), and physically or chemically cross-linked shape-memory multinetwork and composites as multifunctional materials (type III).

Since there are many types of polymers that have the ability to change their shape when triggered by external stimuli, the definition of ‘shape memory’ is often confusing. Behl and Lendlein, who are authorities in the field of SMP systems, have propounded two general categories of such materials, namely, ‘shape-memory’ and ‘shape-changing’ [38, 39]. In this review, we focused on ‘shape-memory’ in a polymer system according to their definition. Because the shape-memory effect in SMPs is not related to a specific material property of single polymers, the control of molecular structure and architecture is particularly important for the functions of SMPs. For the thermally induced SMPs, the mechanism of shape-memory effect is based on the formation of a molecular switching domain, which changes the structural properties in response to a temperature change. From these perspectives, the SMP system can be broadly classified into two types of network architecture: (1) a physically cross-linked network (type I) and (2) a covalently cross-linked network (type II) (figure 3). These can also be specifically separated by type of switching temperature (*T*_switch_), that is, *T*_g_ and *T*_m_. This classification, in other words, categorized their differences in fixing mechanism and origin of permanent shapes, which is also the ‘states’ of their architectures. One of the most important characteristics required for SMPs in cell mechanobiology is that they can actuate under biological conditions. It should be also more beneficial if they actuate sharply in a narrow temperature range. Moreover, biocompatibility is necessary to culture cells on them. In this section, the recent progresses of SMP systems based on the above categories are discussed.

### 2.1. Physically cross-linked SMPs (type I)

One of the major types of SMPs is a physically cross-linked glassy polymer (type I) with *T*_g_, which is the temperature of reversible transition from a hard glassy state to a rubbery molten state. Although the physical cross-linking generally results in less structurally stable networks than the chemical cross-linking, this class of SMPs is easily designed by simultaneously introducing both the hard segments, which determine the highest thermal transition (*T*_high_ or *T*_perm_ that is the temperature stabilizing the permanent shape by acting as physical netpoints), and the soft segments, which determine the switching temperature (*T*_switch_) in materials. The shape of materials is maintained at T < *T*_perm_; however, it is more viscous and fluid at T > *T*_perm_. Therefore, *T*_high_ or *T*_perm_ dictates the stable temperature range of SMPs designed by physical cross-linking. From this consideration of molecular properties, a block copolymer system is the most prominent building block of type I SMPs, and this class of SMPs is constructed using conventional thermoplastics technology. Thermoplastic PU elastomers are typical SMPs that can now be produced on an industrial scale by prepolymer methods. Lin and Chen reported on the PU systems with hard segments composed of methylene diphenyl diisocyanate and 1,4-butanediol, and a polyether switching segment composed of poly(tetramethylene oxide) (PTMO), also called poly(tetrahydrofuran) with low molecular weights. By changing the contents of the hard and switching (soft) segments of PUs, the morphology of the phase-separated structure, which depends on the content of the hard segment, also changed [40]. Besides the PU system combined with PTMO, other polymers such as poly(ethylene adipate) [41], poly(propylene oxide), and poly(butylene adipate) [42] have been utilized as building blocks of the switching-segment-forming phase in PU systems.

One the other hand, the use of *T*_m_ as the triggering switch is more favorable because the enthalpy change of the solid-liquid phase transition is much larger than that of a glass-rubber transition. In the 1990s, styrene-butadiene-based SMPs were developed and commercialized by Asahi Kasei Corporation under the trade name Asmer [20, 21]. Asmer is the block or graft copolymer of poly(styrene-block(graft)-butadiene) and physically cross-linked (thermoplastic) shape-memory resins, in which the *T*_m_ was adjusted to 60 °C by controlling the contents of trans-form poly(1,4-butadiene) (PBD) as the switching-segment-forming phase. Owing to the immiscibility between the poly(styrene) (PS) and PBD block, the copolymer phase separates and PS blocks form discontinuous, amorphous microdomain structures. The *T*_m_ of the PBD crystallites represents the *T*_switch_ for the thermally induced shape-memory actuation, that is, it works as the switching (soft) domain in the physically cross-linked SMP network. Another example of this type of SMP is the linear, phase-separated block copolymers made of poly(ethylene terephthalate) and poly(ethylene oxide) [43–46]. On the other hand, PUs with a semicrystalline polymer as the switching (soft) segments can be also classified into this type I. Poly(*∊*-caprolactone) (PCL) is often used as the switching segment forming polymer networks. PCLs with more than one hydroxyl group at the end group, such as PCL-diol, are generally used to incorporate the PCL as switching (soft) segments into the PU network (figure 3(a)). The crystallizable PCL forms the thermally reversible switching segments caused by crystal-amorphous transition, and the temporary shape can be fixed by crystallizing these segments.

### 2.2. Covalently cross-linked SMPs (type II)

The covalently cross-linked SMPs (type II) has attractive characteristics that include an excellent degree of shape recovery afforded by rubbery elasticity, tunable work capacity during recovery, and an absence of molecular slippage between chains seen in physically cross-linked SMP networks. In contrast to the physically cross-linked SMPs (type I), however, it is difficult to reshape the materials once the shape is formed by cross-linking because the permanent (original) shape is covalently fixed. In contrast to the term ‘thermoplastic’ in the case of type I, they are often called thermoset (thermosetting plastic). A typical material of this type (type II), for example, is cross-linked poly(vinyl chloride) (PVC) which can be prepared by thermal dehydrochlorination and subsequent cross-linking of PVC in HCl atmosphere [47]. Other SMPs which are classified into type II (*T*_switch_ = *T*_g_) are those based on renewable natural sources. Li and co-workers extensively studied this type of SMP for the system comprising copolymerization and chemical cross-linking of renewable natural oils with acrylate monomers to obtain copolymer networks. They mainly focused on the SMP networks made of soybean oil as biological oil and various alkenes by cationic copolymerization [48–50]. The copolymer networks with the shape-memory property can also be designed using a combination of a star-shaped reactive polymer and diisocyanate. Alteheld *et al* have developed *T*_g_-based chemically crosslinked SMP networks that were prepared from the star-shaped hydroxyl-telechelic poly(*rac*-lactide-*co*-glycolide) and aliphatic diisocyanates [51]. The advantage of this system is the ease of manipulation of structural variation, which allows for the regulation of *T*_g_ in the range between 48 and 66 °C.

Aside from the glass transition, the melting transition of semicrystalline networks can also be employed to trigger shape recovery, typically giving a sharper recovery event. Semicrystalline rubbers have been favored as shape-memory materials as a result of their superelastic rheological characteristics, fast shape recovery, and flexible modulus at the fixed stage. One classic material of this family of SMPs is chemically cross-linked trans-polyisoprene (TIP) [52], which is a semicrystalline polymer with a *T*_m_ of 67 °C and a degree of crystallinity of approximately 40%, giving a stiffness of about 100 MPa at room temperature. TIP was chemically cross-linked by peroxides, the enzyme for catalyzing the oxidation-reduction reaction, at 145 °C for 30 min to form a three-dimensional network, thus establishing the permanent (primary) shape and creating the superelastic property required for shape recovery above its *T*_m_. On the other hand, Liu *et al* has successfully developed a chemically cross-linked, semicrystalline trans-polyoctenamer (polycyclooctene, PCO) with a trans content of 80%, a *T*_g_ of 270 °C, a *T*_m_ of 58 °C, and a much better thermal stability for shape-memory applications [53]. Using an analogous concept, Lendlein *et al* have developed shape-memory biodegradable polymers by synthesizing and copolymerizing a narrowly dispersed, oligomeric PCL dimethacrylate with *n*-butyl acrylate under UV radiation to yield a multiblock structure (figure 3(b)) [54]. The PCL segments form a crystalline structure as switching segments to fix the secondary shape at low temperature, leaving the *T*_m_ of the PCL segments to control the shape recovery temperature. Meanwhile, the amorphous *n*-butyl acrylate main chains, together with the PCL dimethacrylate as a cross-linker, form a network that gives rise to the permanent shape and provides the excellent shape recovery by a softening effect with its low *T*_g_ (−55 °C). Uto *et al* has been also developing shape-memory PCL substrates by cross-linking tetra-branched PCL with acrylate end groups in the presence of linear PCL telechelic diacrylates [32–34]. The *T*_m_ of cross-linked PCLs proportionally decreased with decreasing tetra-branched PCL content while the relatively high melting enthalpy was maintained. In particular, the sample with 50/50 wt% mixing ratio of tetra-/di-branched PCL had a *T*_m_ of approximately 33 °C. Surprisingly, the transition occurred over a few °C and the shape fixing rate and shape recovery rate were approximately 99 and 90%, respectively. The advantage of this class of material lies with the very sharp melting transition, owing to the uniformity of the crystals generated by PCL segments with almost identical molecular length. Recently, other synthetic methods based on radiation cross-linking have also been proposed to construct biodegradable PCL networks exhibiting excellent shape-memory properties [55–58].

### 2.3. Multiple networks and composites (type III)

Driven by potential applications, new types of SMPs have been developed by combining with different functions that are not directly linked to the shape-memory effect (type III). Examples of such functionality combinations are electrical conductivity and shape-memory effect, change of color and shape-memory effect, or degradability and shape-memory effect [26, 59, 60]. Several approaches have been explored for creating multifunctionality, involving one-component polymer systems as well as multimaterial systems, e.g*.,* combining two polymers or a polymer and inorganic particles. One example of a single macromolecule, in which several functionalities have been integrated into the polymer structure, is polymer networks with hydrolytical degradability [54, 61]. In multimaterial systems, on the other hand, the additional functionality results from the integration of an additional material (e.g*.,* carbon nanotubes) in a polymer network or multiblock copolymer (figure 3(c)). Indeed, the various external stimuli, such as not only heat [30, 62] but also light [60, 63], moisture [64], magnetic field [65], radio frequency [66] and infrared (IR) light [67], can be utilized as the trigger to activate the shape-memory effects. In 2005, Lendlein *et al* first demonstrated the light-induced SMPs [63]. They introduced the cinnamic acid or cinnamylidene acetic acid groups as photoresponsive molecular switches, which are known to undergo efficient photoreversible [2 + 2] cycloaddition when the sample is exposed to an alternating wavelength *λ* > 260 nm, *λ* < 260 nm in this experiment) to achieve the photoresponsive shape-memory effects. This unique characteristics of light-induced SMPs has enabled the remote activation of shape memory. The same group has also developed the magnetoresponsive SMP [65]. The magnetically induced shape-memory effect was achieved using the composites from the physically cross-linked polymer multinetwork (type I), which is formed by PCL as the switching (soft) segment and poly(*p*-dioxanone) as the hard segment, and magnetic nanoparticles, which consisted of an iron(III) oxide core in a silica matrix. In this system, the power of the magnetic field is converted to thermal energy, that is, induction heating of magnetic nanoparticles. On the other hand, Hazelton *et al* developed the radio-frequency-induced SMP using a composite of covalently cross-linked epoxy network (type II SMP) and magneto-electroelastic particles [66]. They demonstrated that radio-frequency energy coupling is strongly dependent on the applied radio frequency and the level of filled magneto-electroelastic particles, and the radio-frequency energy can be used to actuate the shape-memory effect without interfering with the performance of the SMP. By a similar approach using nanocarbon particles, an IR light-active SMP was also developed by Leng *et al* [67]. Because of the high storage modulus and high IR absorption capability of the nanocarbon particles, the better shape-memory effect of these composites was reported. From these results, the multiple-network formation such as that of inter-penetrating polymer network, a blended structure, and a composite with other functional materials should be a very efficient method of obtaining the SMP with multifunctional properties.

## 3. Biomedical applications of SMPs and SMSs

As a novel kind of biomaterial, SMPs currently cover a broad range of applications in biomedical fields. Especially, new developments of SMPs have been promoted as implantable medical devices in minimally invasive surgery, where a compacted device could be passed through a smaller incision and deployed to its full shape once inside the body. Some of the examples are summarized in table 1. The first study and first clinical trial of an SMP stent was the Igaki–Tamai stent, which is a balloon-expandable poly(L-lactide) (PLLA) stent [68]. Maitland’s group, on the other hand, has been developing SMPs as a clot removal device [69]. In their system, the designed microactuator coil can hold a clot, and the designed devices can also be successfully actuated by a laser. Sharp *et al* designed a prototype neuronal probe using a body-temperature-activated SMP microactuator for slow insertion into the brain tissue [70]. In addition, SMPs have recently presented additional potential in the area of MEMS, actuators, self-healing, unique adhesion devices and cell culture platforms. For example, traditional synthetic substrates and matrices for cell culture have proven to be of only limited utility in efforts to understand and control cell behavior, in large part because they fail to capture the dynamic characteristics of *in vivo* environments. In this context, SMPs have emerged as powerful tools for basic cell studies as well as promising biomedical applications. In this section, therefore, recent developments in SMPs and SMSs as dynamic cell culture substrates are discussed. In particular, the author focuses on the mechanostructural stimuli such as topology as the cues against adhered cells on the substrate to manipulate and regulate cellular functions and fate.

**Table 1.** SMPs for biomedical applications.

Proposed Application	Type of material(s)	Reference(s)
Vascular stent	Poly(L-lactide) (PLLA)	[68]
	Polyurethane (PU)	[90, 91]
	*tert*-Butyl acrylate (*t*-BA)and diethyleneglycol diacrylate (PEGDMA)	[92, 93]
Clot-removal device	PU	[69, 94]
Cardiac valve	PU	[95, 96]
Neural prostheses	Epoxy-based polymer	[70]
Suture	PCL	[26]
Orthodontics	Poly(norbornene)	[97]
Cell culture	PCL	[35–37, 80–83, 98]
	NOA-63	[81]
Bio-MEMS	PCL	[27–29]

### 3.1. Effects of mechanostructural cues on cell functions

**Figure 4.**
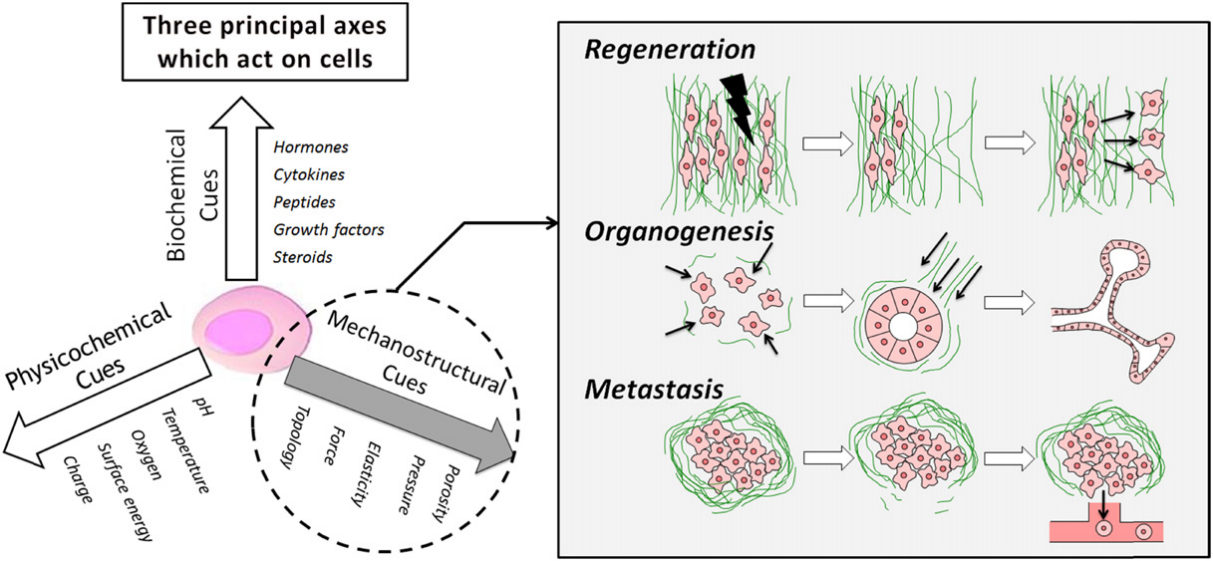
The tripartite axes of cues which act on cells. These factors influence the orchestration of cellular function such as tissue regeneration, organogenesis, cancer metastasis etc.

Recent reports have revealed that many types of cells have the capability of sensing and reacting to the surrounding cues of the ECM. More recently, the cell–ECM interactions are considered to be important extrinsic factors that regulate cell fate because determination of cellular phenotypes is considered to be governed by a complex set of extrinsic cues in collaboration with intrinsic gene regulatory machinery [71, 72]. Figure 4 shows three principal axes which act on cells. Among those extrinsic cues, an increasing number of studies have shown that the mechanostructural stimuli such as elasticity, topography and mechanical force alter cell migration, over all morphology, the structure of the cytoskeleton, expression of specific genes, as well as the lineage of stem cell differentiation [73]. For example, stiff gels promote spreading and scattering of adherent cells, while soft gels promote soft tissue differentiation and tissue-like cell–cell associations. In addition, adherent cells migrate preferentially toward stiffer regions. Engler *et al* first reported that differentiation of mesenchymal stem cells (MSCs) is highly sensitive to substrate stiffness [74].

In addition to sensing stiffness, topographical cues also play an integral role in influencing cell fate. Teixeira *et al*, for example, reported the importance of substrate micro- and nano-topography on the orientation of the cytoskeleton and cells themselves through the organization and regulation of focal adhesions [75]. Cukierman *et al* demonstrated a major discrepancy between 2D and 3D materials in that fibroblast readily migrated along ligand-dense ECM fibers in 3D at rates ∼1.5 times faster than in 2D system [76]. Kim *et al* have developed an engineered myocardial tissue that mimics the structural and functional properties of native myocardial tissue specifically the underlying ECM architecture [77]. They found that the engineered tissue structure and function were highly sensitive to variation of the nanoscale topographic features of the substratum. They also reported that cells can recognize the variation of topographic pattern density and anisotropy, resulting in migration toward the denser area from their initial positions [78]. Furthermore, micro- and nano-topographies can also be used not only for the differentiation, but also for the maintenance of stemness via the suitable design of geometry. Chen *et al* prepared glass substrates having nanoscale roughness (1, 70, and 150 nm) by reactive ion etching technique. When human embryonic stem cells (hESCs) were cultured on these substrates, hESCs on the smooth surface (roughness of 1 nm) maintained stemness in long term culture of seven days, while those on rough surfaces spontaneously differentiated [79]. Furthermore, stemness was decreased as the roughness increases from 1 to 70 and 150 nm (93.6, 41 and 36.6%, respectively). These studies indicate that the manipulation of topographical cues has strong potential as a strategy for stem cell engineering and regenerative medicine by mimicking changing physiological conditions during would healing, organogenesis, and cancer metastasis.

### 3.2. SMSs for directing cell fate

As mentioned above, cell responsiveness to their surrounding mechanical cues has been the subject of active research. In spite of a considerable amount of ongoing research, however, current efforts are centered on rather static patterns. Due to the dynamic nature of the regeneration processes, static substrates seem to be deficient in mimicking changing physiological conditions, such as development, wound healing and disease. Therefore, the scientific community has recently shown increased interest in developing surfaces with tunable abilities. In this context, SMPs or SMSs have emerged as powerful tools for basic cell studies as well as promising biomedical applications. Neuss *et al* were the first to demonstrate the bulk shape-memory effect on adhered cells [80]. They characterized the behaviors of L929 mouse fibroblasts, human MSCs, human mesothelial cells, and rat mesothelial cells on a PCL-based SMP network. They synthesized the PCL networks by radical polymerization of PCL-dimethacrylate, and showed a *T*_m_ of 52 °C. They determined the shape-memory effect on adhered L929 cells upon activation by a 10 s heating incubation at 54 °C. After the activation of the shape-memory effect, the cell monolayer was disconnected and some cells underwent apoptosis due to generated shear forces at different points on the surface. Note that heating itself did not affect cell viability.

The first example of the ‘surface’ shape-memory effect applied to a dynamic cell culture substrate was shown by Davis *et al* [81]. To enable surface shape-memory during cell culture, they prepared a glassy SMS from Norland Optical Adhesive 63 (NOA-63) because the recovery temperature can be tuned by manipulating the cross-linking condition in this system. After plating the cells on the substrate with temporal grooves, they triggered shape-memory activation using a change in temperature tailored to be compatible with mammalian cells such as mouse embryonic fibroblasts, thereby causing a topographic transformation back to the original flat surface. They found that the programmed erasure of substrate topography caused a decrease in cell alignment as evidenced by an increase in angular dispersion with corresponding remodeling of the actin cytoskeleton. Cell viability remained higher than 95% before and after topography change and temperature increase. However, the large, irregular dimensions of the surface patterns limited the degree of control over fibroblast cell morphology. From this regard, Le *et al* developed biocompatible SMSs that can accommodate diverse, well-defined, and biologically relevant surface transformations under physiological conditions [82]. This dynamic cell culture substrate was prepared by crosslinking a methacrylate end-functionalized PCL macromonomer in a mold to produce a permanent shape and then mechanically compressed the desired shape to form a temporary shape. These substrates showed the potential to transition between two predefined surface topography by changing the temperature from 28 to 40 °C. In fact, hMSC morphology switched from highly aligned to stellate shaped in response to a surface transformation between a 3 × 5 *μ*m groove array and planar surface.

**Figure 5.**
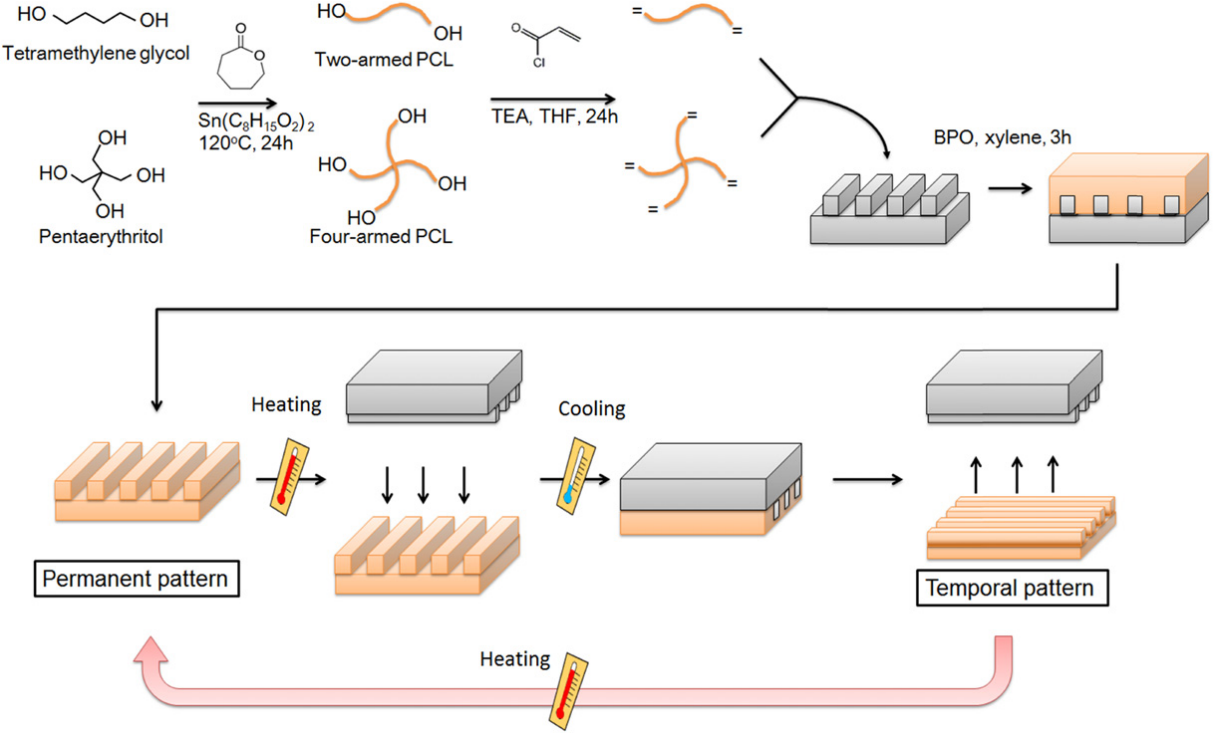
Fabrication of shape-memory nanotopographic surfaces. Two-branched and four-branched PCL are synthesized by a CL ring-opening polymerization. Acryloyl chloride is then reacted with the end of the branched chains. To memorize ‘permanent surface patterns’, PCL macromonomers are cured on a nanopatterned mold. To program ‘temporal surface patterns’, the films are compressed in a thermo chamber above the *T*_m_. Application of heat transitions temporary surface patterns to permanent patterns. TEA: triethyleneamine, THF: tetrahydrofuran, BPO: benzoyl peroxide. Modified from figure 1 in [37].

**Figure 6.**
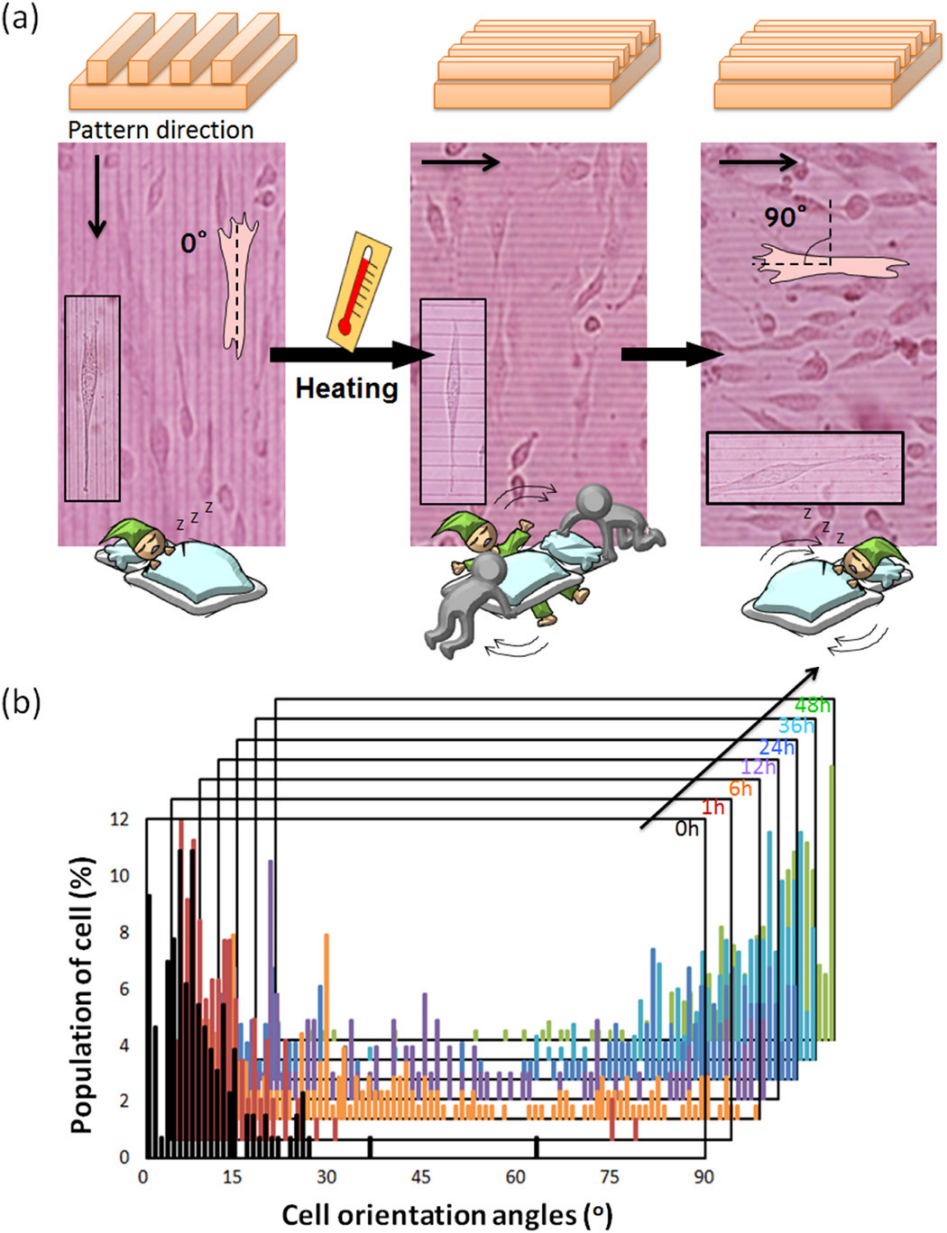
(a) Phase contrast images of NIH 3T3 fibroblasts seeded on the PCL films with the temporal grooved surface (left). For the surface shape-memory experiment, the cells were subjected to a 37 °C heat treatment for 1 h (middle). The cells were then allowed to equilibrate at 32 °C for 48 h (right). (b) Histograms of cell orientation angle on the PCL film before and after shape-memory activation. Cell orientation angles were defined as the angle against the temporary groove direction. Combined and modified from figures 1, 4, and 5 in [36].

**Figure 7.**
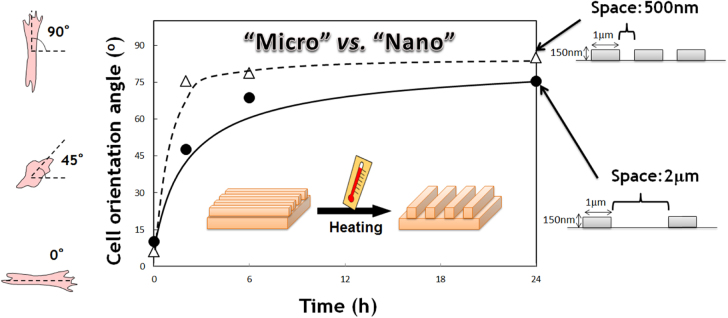
Time-dependent changes in the orientation angles of cells on the PCL film after shape-memory transition from a temporal grooved pattern to the permanent grooved patterns which is perpendicular to the original shape.

The author’s group has also been developing a SMS system with dynamically tunable nanopatterns to direct cell fate [35]. The shape-memory nanopatterns were prepared by chemically crosslinking semi-crystalline PCL in a mold to show shape-memory effects over its melting temperature (*T*_m_ = 33 °C). Permanent surface patterns were first generated by crosslinking the PCL macromonomers in a mold, and temporary surface patterns were then embossed onto the permanent patterns (figure 5). The temporary surface patterns could be easily triggered to transition quickly to the permanent surface patterns by a 37 °C heat treatment. One of the great advantages of PCL over other temperature-responsive polymers is that surface properties such as wettability and charge are independent of temperature. Using this substrate, we have successfully demonstrated time-dependent cell orientation changes by inducing nanotopographical transition from grooves with a height of 300 nm to flat. Upon transition from the grooved topography to a flat surface, cell alignment was lost and random cell migration and growth ensued. We have also succeeded in inducing a 90° rotation of the cell orientation by using shape-memory nanogrooves, the direction of which was transitioned 90° to the temporary grooves (figure 6) [36]. Interestingly, 90% of cells did not change their direction 1 h after the topographic transition. By 36 h, however, 70% of cells realigned parallel to the permanent grooves that emerged. To understand the effects of pattern dimension (nm versus *μ*m) on the interlude between the topographic transition of shape-memory nanopatterns and cell response on them, we have also monitored time-dependent changes in surface nanotopographic features associated with shape-memory transition as well as the cell morphology or alignment [37]. Holographic microscope revealed that the application of heat to PCL SMP quickly and completely transitioned temporary surface patterns substrate to permanent patterns within 30 s. However, it took more than 2 and 8 h for cells on substrate with 500 and 2000 nm grooves to induce 90°-rotation of the cell orientation, respectively (figure 7). This different alignment behavior can be explained by the different adhesion strength and reorganization of cytoskeletal proteins on nano versus micropatterns. To our best knowledge, we first revealed that dynamic control of geometrical shape exert a dramatic effect on the realignment of adhered cells even using the same material.

**Figure 8.**
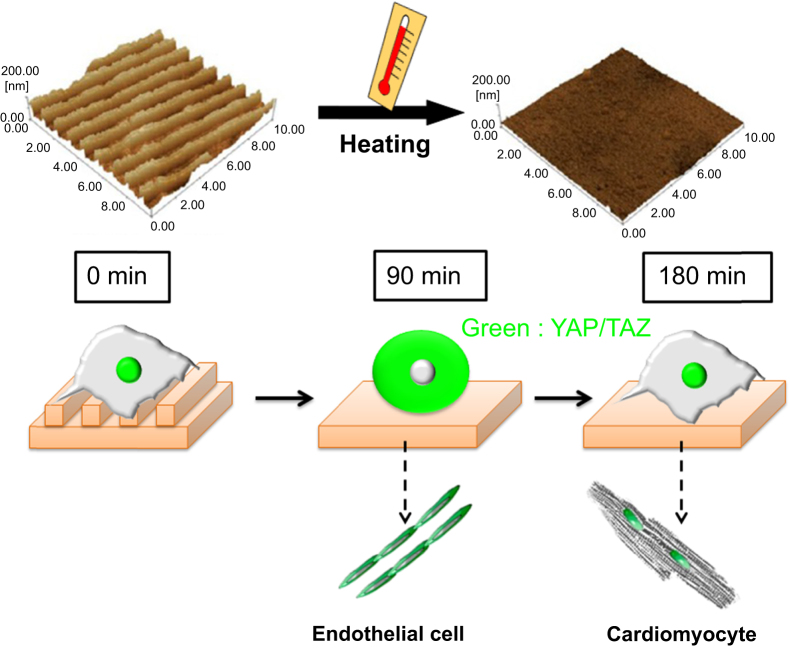
YAP/TAZ nuclear localization is sensitive to dynamic changes in substrate nanopattern in cardiac progenitor cells. PCL-based thermo-responsive substrates having shape-memory effect were produced to display a temporary nanopattern at 32 °C. The surfaces could be induced to a thermal transition after switching the temperature to 37 °C to acquire a permanent flat surface. When shape-memory transition is activated, cells are displaced from the surface and encounter a dramatic but transient switch in YAP/TAZ nuclear expression, which is restored after 180 min.

These dynamic changes in nanotopography created on PCL substrate can also influence the intracellular localization of YAP/TAZ in cardiac progenitor cells [83]. YAP and TAZ are respectively Yes-associated protein YAP and WW domain-containing transcription regulator protein 1 which are the downstream effectors of the Hippo pathway. The YAP/TAZ subcellular localization, strictly regulated by cell–cell and cell–ECM interaction, has been correlated to cell mechanosensing. Following surface change from nanotopography (300 nm of line ridge, 500 nm of groove, 120 nm of height) to flat by switching temperature to 32–37 °C, a significant decrease in nuclear expression of YAP/TAZ could be detected after 90 min. However, the percentage of YAP nuclear positive cells returned to the original values after 180 min (figure 8). This result indicates that the YAP/TAZ transcriptional coactivators may act as intracellular mechanical rheostats mediating the effectors of mechanical dosing on stem cell plasticity by a persistent presence in the nucleus. In the future, these approaches will also enable not only unprecedented observations of time-dependent cell–substrate interactions without the need for invasive forces against intact adhered cells but also direct manipulation of cell function and fate.

### 3.3. Spatio-temporal control of cell fate on SMSs

**Figure 9.**
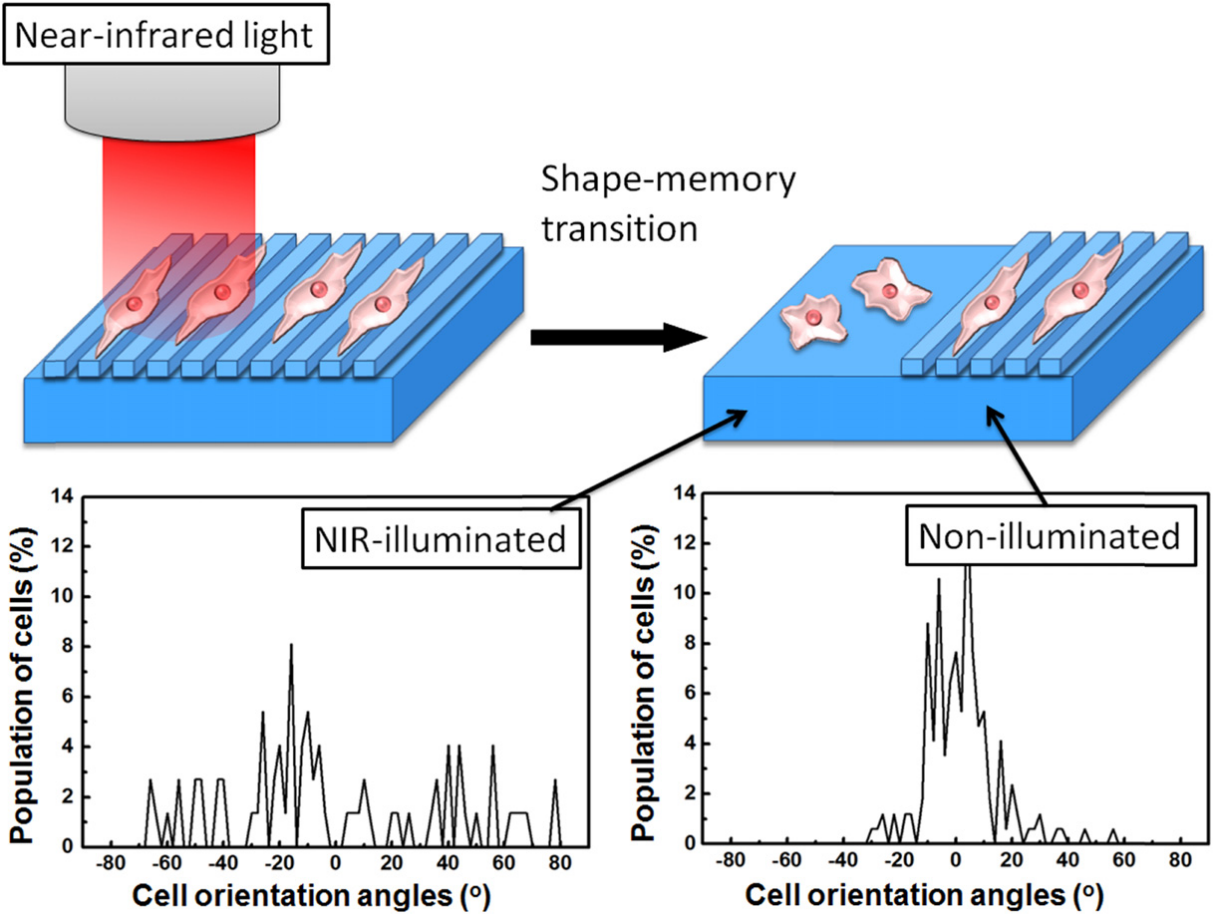
Schematic illustration of near-infrared-irradiation-induced remote activation of surface shape-memory to direct cell orientations. Cells were seeded on the temporal patterned surface and cultured at 37 °C for 3 h. The NIR light (0.8 W cm^−1^) was then irradiated until the surface transition was completed. Histograms show the cell orientation angle against pattern direction in non-illuminated (right) and photo-illuminated regions (left). Modified from figure 7 in [89].

Although a thermally induced shape-memory effect can be achieved by an increase in temperature, shape-memory activation by remote light irradiation could lead to a variety of potential biomedical or other applications. From this regard, we have recently demonstrated the light-induced shape-memory transitions of nanopatterns using gold nanorods (AuNR)-embedded PCL films [84]. AuNRs have been widely employed in numerous biomedical applications, including hyperthermia therapy and biological sensing, due to their photothermal effects [85, 86]. One of the advantages is that the surface plasmon resonance extinction of AuNRs in the near-IR (NIR) region (650–900 nm) provides opportunities for NIR photoabsorption and scattering, in which region there is very limited absorption for most biological tissues, including hemoglobin and water. There have been several reports on remote-controllable SMPs using AuNRs [87, 88]. We have extended this photothermal effect of AuNRs to the local and remote activation of nanopatterns by spatially limiting the NIR-illuminated region. First, the NIR-induced shape-memory nanopatterns are prepared by chemically crosslinking PCL in the presence of AuNRs. Exposure to NIR light can successfully induce the photothermal heating of embedded AuNRs and consequently, the shape-switching transition to permanent patterns over the melting temperature of PCL. The shape-memory transition is spatially limited to the photo-illuminated region, depending on the concentration of embedded AuNRs, the intensity of the NIR light, or the irradiation time. Cell alignment in the NIR-irradiated region was lost and random cell migration was ensured because it caused the surface transition to a flat surface, while cells on the non-illuminated region were still aligned along the direction of the temporal pattern (figure 9) [89]. This finding is novel in that it is the first study that controls the cell orientation locally and remotely on the SMSs by NIR irradiation. The NIR-responsive shape-memory system offers significant promise for the creation of topographically tunable substrates because of their remote-capability to undergo large elastic deformations and to rapidly return to their initial undeformed state.

## 4. Summary

As described above, cell responsiveness to their surrounding mechanical cues (especially nano-topography) has been an active research subject. Although our understanding of biochemical and physicochemical cues in the cellular microenvironment has been constantly improved, these sophisticated hierarchical interactions have often been overlooked when experiments were conducted in the context of static system. In this regards, SMPs or SMSs are becoming one of the most powerful tools available to lead to a new generation of dynamic systems that are capable of responding to external stimuli or cellular signals with spatial precision. From the numerous investigations described herein and elsewhere, it is clear that dynamically tunable materials play a critical role in regulating cell behavior. Scientists should now be able to take full advantage of SMPs or SMSs to gain additional insight into fundamental mechanisms driving cell behavior. New developments of independent, dynamic regulation of multiple parameters during tissue formation are desired. In addition, development of dynamic materials capable of incorporating a wide range of biologically relevant molecules has the potential to provide useful therapeutic benefits because it has been difficult for conventional technologies to produce highly-organized constructs with tunable properties. Since an ongoing challenge is to control stem cell differentiation, novel nanoatchitectonics-based strategies for substrate design will facilitate the creation of new classes of dynamic substrates in the future.
